# Pulmonary hypertension among patients hospitalized with acute heart failure in Buea, South West Region of Cameroon

**DOI:** 10.11604/pamj.2022.42.216.33076

**Published:** 2022-07-20

**Authors:** Clovis Nkoke, Ahmadou Musa Jingi, Jean Jacques Noubiap, Anastase Dzudie

**Affiliations:** 1 Faculty of Health Sciences, University of Buea, Buea, Cameroon,; 2 Clinical Research Education, Networking and Consultancy, Douala, Cameroon,; 3 Faculty of Health Sciences, University of Bamenda, Bamenda, Cameroon,; 4 Centre for Heart Rhythm disorders, South Australian Health and Medical Research Institute, University of Adelaide and Royal Adelaide Hospital, Adelaide, Australia,; 5 Faculty of Medicine and Biomedical Sciences, University of Yaounde 1, Yaounde, Cameroon

**Keywords:** Heart failure, pulmonary hypertension, outcome

## Abstract

**Introduction:**

pulmonary hypertension (PH) is a common and severe complication in patients with heart failure (HF). It is associated with increased morbidity and mortality. There is limited data in Cameroon on the prevalence of PH in hospitalized HF patients and its impact on outcome.

**Methods:**

we analyzed data from consecutive adult patients hospitalized with. Pulmonary hypertension (PH) was defined as pulmonary artery systolic pressure (PASP) ≥ 35mmHg.

**Results:**

eighty-six (86) consecutive patients were hospitalized and 66(76.7%) had measurable PASP on echocardiography. Of those with echocardiographically measurable PASP (66), there were 39 (59.1%) females. The median (IQR) age was 60 (42-76) years. The prevalence of PH was 93.9%. PH was present in all (100%) patients with right heart failure (RHF) and in 62 (93.9%) patients with left heart failure (LHF). Severe PH (PASP ≥55 mmHg) was seen in 45 (68.2%, [95% CI: 55.6-75.1]) patients. The mean PASP was significantly higher in those with isolated RHF compared with those who had isolated left or bi-ventricular failure. Factors likely associated with moderate-to-severe PH (PASP ≥ 45 mmHg) were female sex, RHF, and right atrial dilatation. After adjusting for sex, right atrial dilation was independently associated with moderate-to-severe PH. In-hospital death occurred in 7 (10.6%, [95% CI: 4.4-20.6]) patients. The median (IQR) time to death was 6 (3-7) days and ranged from 2 to 8 days. All deaths (100%) occurred in those with moderate-to-severe PH.

**Conclusion:**

the prevalence of pulmonary hypertension in hospitalized heart failure patients was high with two third of the patients having severe PH, and most commonly occurred in females. All deaths occurred in patients with moderate-to-severe PH.

## Introduction

Heart failure (HF) and pulmonary hypertension (PH) are two commonly encountered clinical conditions in sub-Saharan Africa and they are associated with high morbidity and mortality [1-4]. In recent decades, there has been an increase in the diagnosis of PH and it is estimated that about 80% of the global burden of PH occurs in low and middle-income countries [5]. Heart failure accounts for up to 42.5% of medical admissions in sub-Saharan Africa (SSA) and its prevalence is expected to rise [6]. Pulmonary hypertension is highly prevalent and is a well-established complication of heart failure [7,8]. Overall, PH is estimated to occur in 60% of heart failure cases [9,10]. It was reported in up to 66.7% and 70.4% of patients admitted and followed up for heart failure respectively in Nigeria [11,12]. Left heart diseases (LHD) are the leading causes of pulmonary hypertension globally [13-15]. In SSA, left heart failure was responsible for 69% of cases of PH in the PAPUCO registry [16].

Overall, PH is associated with increased morbidity and mortality in heart failure patients [17]. Although heart failure is a frequent cause of hospital admission in Cameroon, the prevalence of PH and outcomes in patients hospitalized with heart failure in Cameroon are unclear. This study aimed to determine the prevalence of PH, clinical characteristics, and the impact on outcome in a heterogeneous cohort of patients hospitalized for heart failure in the South West region of Cameroon.

## Methods

**Study design and setting:** between June 2016 and November 2017, we carried out a cross-sectional descriptive and analytic study in the Buea Regional Hospital. This is a secondary level Hospital and serves as one of the two main referral centres in the region, with a bed capacity of 111 beds, and a catchment population of about 200,000 inhabitants. The Hospital also serves as one of the teaching hospitals of the University of Buea. Facilities for cardiac evaluation at the centre include; chest radiography, 12-lead electrocardiograph, and echocardiography.

**Study population:** these were adults of both sexes aged ≥ 18 years who were hospitalized for heart failure during the study period. The participants were prospectively recruited.

**Procedure and measurements:** the methods involved in this study have been previously described [18]. We collected demographic data (age and sex), and medical history (previous diagnosis of heart failure, hypertension, diabetes, alcohol consumption, tobacco use, atrial fibrillation). Each patient underwent a complete clinical evaluation for symptoms and signs of heart failure. We measured the blood pressure according to standard procedures and blood was collected for biochemical analysis (serum creatinine, haemoglobin, sodium, potassium, and fasting blood glucose). Each patient then underwent a 12-lead resting ECG and a comprehensive cardiac ultrasound by a trained Cardiologist (CN) with a Sonoscape S8 ultrasound machine (Sonoscape China). Echocardiography was obtained within 48 hours of admission as much as possible.

**Outcome variable:** the outcome variable was pulmonary hypertension (PH). Pulmonary hypertension was defined as mean pulmonary artery systolic pressure ≥ 35mmHg (PASP ≥ 35 mmHg) (16). Pulmonary artery pressure was measured using the tricuspid regurgitant jet. Pulmonary artery systolic pressure (PASP) was estimated by summing the peak systolic trans-tricuspid pressure gradient calculated from peak velocity of TR and right atrial pressure (RAP) estimated by diameter and inspiratory collapsibility of inferior vena cava (IVC). We determined the prevalence of PH in patients with measurable pulmonary artery systolic pressure.

**Sample size and statistical analyses:** a convenient sample of all patients was considered for this study. Data were analyzed using Epi Info version 7. We have presented continuous variables as means (SD) or median (IQR) where appropriate. We have presented discrete variables as frequencies and proportions (95% confidence interval). We have compared means with ANOVA statistics and proportions with Chi-squared or Fischer exact test where appropriate. A p-value < 0.05 was considered statistically significant.

**Ethical considerations:** this study was approved by the administrative authority of the Buea Regional Hospital acting as the local ethics committee. All participants or their legal guardians provided written informed consent to be included in the heart failure registry. All the patients approached accepted to be included in the registry.

## Results

### General characteristics

A total of 86 patients with heart failure were hospitalized during the study period. The pulmonary artery systolic pressure (PASP) was echocardiographically measurable in 66 (76.7%) patients. Of those with echocardiographically measurable PASP (n=66), there were 27 (40.9%: [95% CI: 29-53.7]) males and 39 (59.1%: [95% CI: 46.3-71.1]) females. The median (IQR) age was 60 (42-76) years and ranged from 20 to 100 years. There was no significant difference between the sexes.

### Clinical and echocardiographic characteristics of patients with measurable and non-measurable pulmonary artery systolic pressure

The clinical characteristics of patients with measurable and non-measurable PASP are shown in Table 1. Patients with measurable PASP had significantly lower blood pressures. The echocardiographic characteristics of patients with measurable and non-measurable PASP are shown in Table 2. Patients with measurable PASP had significantly lower E-wave deceleration time, higher E/A ratio, higher rates of RA dilation, and higher rates of bi-ventricular failure. The most frequent echocardiographic anomaly was left atrial dilation in 52 (78.8%, [95% CI: 67-89.9]) patients. Bi-ventricular failure was the most frequent in 39 (59.1%, [95% CI: 46.3-71.1]). Isolated Right Heart Failure was seen in 10 (15.2%, [95% CI: 7.5-26.1]) patients (Table 2).

**Table 1:** clinical characteristics of patients with echocardiographically measured pulmonary pressure (n=66) compared with those with unmeasured pulmonary pressure (n=20)

Variable	Overall (n=86)	Pulmonary pressure measurable with Echo		p-value
		Yes (n=66)	No (n=20)	
Age (years), mean (SD)	59.4 (18.3)	58.7 (19.3)	61.6 (15)	0.563
Male sex, n(%)	38 (42.2)	27 (40.9)	11 (55)	0.266
**Medical History, n(%)**				
Chronic Heart Failure	13 (15.1)	11 (16.7)	2 (10)	0.466
Hypertension	46 (53.3)	33 (50)	13 (65)	0.239
Diabetes	12 (14)	7 (10.6)	5 (25)	0.104
Current smoking	6 (7)	5 (7.6)	1 (5)	0.92
Atrial Fibrillation	2 (2.3)	2 (3)	0 (0)	0.431
Chronic Kidney Disease	5 (5.8)	3 (4.5)	2 (10)	0.361
Alcohol consumption	9 (10.5)	8 (12.1)	1 (5)	0.362
**Symptoms**				
NYHA Class				
II	5 (5.8)	3 (4.5)	2 (10)	
III	44 (51.2)	32 (48.5)	12 (60)	0.331
IV	37 (43)	31 (47)	6 (30)	
Fatigue	86 (100)	66 (100)	20 (100)	NA
Orthopnea	82 (95.3)	64 (97)	18 (90)	0.195
**Physical findings**				
SBP, mean (SD)	140.3 (35.4)	134.6 (29.6)	157.3 (45.8)	0.006
DBP, mean (SD)	92.5 (27.8)	89.2 (23.4)	103.2 (45.8)	0.048
HR, mean (SD)	96.2 (21.9)	96.1 (24.3)	96.7 (11.5)	0.923
Pedaledema, n(%)	77 (89.5)	61 (92.4)	16 (80)	0.112
Rales, n (%)	65 (75.6)	52 (78.8)	13 (65)	0.209

**Table 2:** echocardiographic characteristics of patients with echocardiographically measured pulmonary pressure (n=66) compared with those with unmeasured pulmonary pressure (n=20)

Variable	Overall (n=86)	Pulmonary pressure measurable with Echo		p-value
		Yes (n=66)	No (n=20)	
**Mean (SD) values**				
Septum (mm)	10.4 (2.9)	10.1 (2.7)	11.4 (3.3)	0.089
Posterior wall (mm)	10.3 (2.5)	10.1 (2.3)	10.95 (3.2)	0.218
LV End-diastolic diameter (mm)	56.96 (11.2)	56.6 (11.9)	58.2 (8.6)	0.563
LV End-systolic diameter (mm)	45.7 (13.4)	45.4 (13.8)	46.4 (12.6)	0.774
Relative Wall Thickness	0.39 (0.16)	0.39 (0.17)	0.39 (0.14)	0.842
LV Mass (g)	243.4 (97)	232.9 (92.1)	277.9 (107)	0.069
LV Ejection fraction (%)	39.4 (19.3)	38.6 (19.4)	41.95 (19.1)	0.502
LV Fractional shortening (%)	20.2 (11.9)	19.9 (12.1)	21.2 (11.4)	0.678
LAArea (cm^2^)	24.2 (6.9)	24.5 (6.8)	23.2 (7.3)	0.460
LA Diameter (mm)	41.4 (8.1)	41.4 (8)	41.7 (8.5)	0.853
RA Area (cm^2^)	20.8 (6.9)	21.4 (6.9)	18.5 (6.6)	0.101
TAPSE	15.4 (2.3)	15.1 (2.4)	16.1 (1.8)	0.115
E-wave Deceleration time (ms)	117.6 (35.2)	113 (29.8)	131.95 (46.4)	0.035
E/A ratio	1.9 (0.9)	2.04 (0.95)	1.4 (0.7)	0.013
**Proportion, n(%) values**				
LV Hypertrophy	39 (45.3)	29 (43.9)	10(50)	0.812
LA Dilation	65 (75.6)	52 (78.8)	13 (65)	0.209
RA Dilation	47 (54.7)	40 (60.6)	7 (35)	0.034
Type of HF syndrome				
Left Ventricular Failure	28 (32.6)	17 (25.8)	11 (55)	
Right Ventricular Failure	13 (15.1)	10 (15.2)	3 (15)	0.034
Bi-ventricular Failure	45 (52.3)	39 (59.1)	6 (30)	
TAPSE < 17mm	58 (67.4)	49 (74.2)	9 (45)	0.014

### Prevalence of pulmonary hypertension

Pulmonary Hypertension (PASP = 35 mmHg) was present in 62 (93.9%, [95% CI: 85.2-98.3]) patients. This was seen in all (100%) patients with right heart failure and 62 (93.9%) patients with left heart failure (Table 3). Severe PASP (PASP = 55 mmHg) was seen in 45 (68.2%, [95% CI: 55.6-75.1]) patients. The mean PASP was significantly higher in those with isolated right heart failure compared with those who had isolated Left or bi-ventricular Heart Failure (Table 3). The mean PASP was comparable in those with HFpEF, HFmrEF, and HFrEF (Table 4). Hypertensive heart disease (43.5%), Cor pulmonale (16.1%), and rheumatic heart disease (8.1%) were the most frequent causes of heart failure in patients with PH (Table 5). Factors likely associated with moderate-to-severe pulmonary hypertension (PASP = 45 mmHg) were female sex, right heart failure, and right atrial dilation (Table 6). After adjusting for sex, right atrial dilation was independently associated with moderate-to-severe pulmonary hypertension (aOR: 6.7, CI: 1.3-50.4). Right ventricular dysfunction (TAPSE < 17mm) was not associated with the severity of pulmonary hypertension (aOR: 2.8, [95% CI: 0.5-13.4]).

**Table 3:** pulmonary pressure in patients with left heart failure, right heart failure, or both (n=66)

Variable	Overall (n=66)	Type of heart failure syndrome			p-value
		LHF + RHF (n=39)	LHF (n=17)	RHF (n=10)	
Mean (SD) PASP, mmHg	64.1 (20.2)	60.3 (17.1)	59.5 (19.6)	86.8 (22.3)	<0.001
Median (IQR) PASP, mmHg	61 (52-73)	60 (52-70)	63 (39-75)	90.5 (76-100)	
PASP range (mmHg), n(%)					
<35	4 (6.1)	2 (5.1)	2 (11.8)	0 (0)	0.679
35-44	8 (12.1)	5 (12.8)	3 (17.7)	0 (0)	
45-54	9 (13.6)	6 (15.4)	2 (11.8)	1 (10)	
≥55	45 (68.2)	26 (66.7)	10 (58.8)	9 (90)	

**Table 4:** pulmonary pressure in heart failure patients with reduced, mid-range, and preserved left ventricular Ejection Fraction (n=57)

Variable	Overall (n=57)	Type of left heart failure			p-value
		HFrEF (n=38)	HFmrEF (n=8)	HFpEF (n=10)	
Mean (SD) PASP, mmHg	60.3 (17)	60.3 (15.7)	58 (19.9)	61 (24.2)	NS
Median (IQR) PASP, mmHg	60 (51-71)	60 (52-71)	60.5 (41-67.5)	60 (41-63)	
PASP range (mmHg), n(%)					
<35	4 (7)	2 (5.3)	2 (25)	0 (0)	0.089
35-44	8 (14)	5 (13.2)	0 (0)	3 (30)	
45-54	8 (14)	7 (18.4)	1 (12.5)	0 (0)	
≥55	37 (64.9)	24 (63.2)	5 (62.5)	8 (70)	

**Table 5:** etiologies of heart failure in patients with pulmonary hypertension (n=62)

Etiologies of heart failure	Pulmonary hypertension (n=62)		Overall, n (%)
	Type 2, n (%)	Types 1, 3, 4, 5, n (%)	
Rheumatic Heart Disease	5 (9.6)	0 (0)	5 (8.1)
Hypertensive Heart Disease	27 (51.9)	0 (0)	27 (43.5)
Idiopathic Dilated cardiomyopathy	7 (13.5)	0 (0)	7 (11.3)
Ischemic Heart Disease	4 (7.7)	0 (0)	4 (6.5)
HIV Cardiomyopathy	2 (3.8)	0 (0)	2 (3.2)
Cor pulmonale*	0 (0)	10 (100)	10 (16.1)
Others	5 (9.6)	0 (0)	5 (8.1)
Peripartum Cardiomyopathy	2 (3.8)	0 (0)	2 (3.2)
Total	52 (100)	10 (100)	62 (100)

**Table 6:** factors associated with moderate-to- severe pulmonary hypertension (PASP ≥ 45 mmHg) (n=55)

Variable	PASP ≥ 45mmHg, n (%)	Odds ratio (95% CI)	p-value
Female sex			
Yes	35 (89.7)	3.1 (0.8 -11.8)	0.093
No	20 (74.1)	1	
Age > 50 years			
Yes	34 (79.1)	0.36 (0.1-1.8)	0.204
No	21 (91.3)	1	
Systolic BP ≥ 140 mmHg			
Yes	18 (72)	0.3 (0.1-1.1)	0.054
No	37 (90.2)	1	
Diastolic BP ≥ 90 mmHg			
Yes	21 (80.8)	0.7 (0.2-2.7)	0.652
No	34 (85)	1	
NYHA Class IV			
Yes	25 (80.6)	0.69 (0.2-2.6)	0.581
No	30 (85.7)	1	
LVHypertrophy			
Yes	39 (83)	0.91 (0.2-3.9)	0.903
No	16 (84.2)	1	
LVEF < 40%			
Yes	32 (84.2)	1.46 (0.3-4.2)	0.824
No	23 (82.1)	1	
LV Area > 20 cm^2^			
Yes	41 (78.8)	NA	0.102
No	14 (100)		
Type 3 diastolic dysfunction			
Yes	42 (80.8)	0.32 (0.04-2.8)	0.281
No	13 (92.9)	1	
TAPSE < 17mm			
Yes	43 (87.9)	2.99 (0.8-11.5)	0.102
No	12 (70.6)	1	
RA Area > 18 cm^2^			
Yes	37 (92.5)	5.5 (1.3-23.2)	0.013
No	18 (69.2)	1	

### Outcome

The median (IQR) duration of hospitalization was 7 (6-10) days and ranged from 2 to 21 days. Hospital stay > 7 days was seen in 28 (42.4%, [95% CI: 30.3-55.2]) patients. Moderate-to-severe pulmonary hypertension was not associated with hospital stay > 7 days (OR: 0.4, [95% CI: 0.1-1.4], p=0.182). In-hospital death occurred in 7 (10.6%, [95% CI: 4.4-20.6]) patients. The median (IQR) time to death was 6 (3-7) days and ranged from 2 to 8 days. All deaths (100%) occurred in those with moderate-to-severe pulmonary hypertension.

## Discussion

This analysis aimed to determine the prevalence of pulmonary hypertension (PH) in patients hospitalized with heart failure, its clinical characteristics, and its impact on the outcome. The prevalence of PH was 93.9% in patients with measurable pulmonary artery pressure with two out of three patients having severe PH. The most common causes of heart failure in patients with PH were hypertensive heart disease and cor pulmonale. All deaths (100%) occurred in patients with moderate-to-severe pulmonary hypertension.

In the Cameroonian population, there is very limited data on PH in heart failure patients. Patients in our study were older than that reported in Nigeria (59.4 vs 45.9 years) in a study of pulmonary hypertension among patients hospitalized with heart failure [11]. This can be explained by a higher prevalence of hypertensive heart failure in our study. However, there were a similar proportion of females in both studies with females representing more than half of the total population [11]. This was similar to that reported in other studies [19]. The prevalence of pulmonary hypertension in heart failure patients reported in our study was higher than that reported by previous studies in neighbouring Nigeria with the prevalence of PH of 66.3% and 70.4% among patients with heart failure [11,12]. The prevalence was also higher than that reported in America with a prevalence of PH of 79% in HF patients in a community study [20]. This difference could be explained by differences in estimated pulmonary pressures, clinical characteristics, and the etiologies of heart failure. Our study was a hospital-based study with patients hospitalized with acute decompensated heart failure contrary to the study by Bursi *et al*. who evaluated patients in the community with some as outpatients [20]. The prevalence of cor pulmonale in our study was 16.1% compared to 6.2% in the study by Karaye *et al*. in Nigeria [11] which can partly explain the higher prevalence of PH in our study. The pulmonary artery pressure used in our study was measured on admission, this was not repeated after decongestion and before discharge when the patient has been stabilized, this can explain the high prevalence of PH in our study. But also, in the study by Karaye *et al*. evaluation was done within 48 hours of admission and it is not clear if the pulmonary artery pressure was re-evaluated when patients were stabilized [11]. In our study, some patients would have had a decrease in pulmonary pressure if they were re-evaluated after decongestion and stabilization.

There was no statistically significant difference in pulmonary hypertension across the different heart failure phenotypes. This was similar to the findings of Amadi *et al*. in Nigeria [12]. Clinically, when comparing patients with measurable pulmonary pressure to those without measurable pulmonary pressure, patients with measurable pulmonary artery pressure had significantly lower systolic and diastolic blood pressures. Karaye *et al*. also reported lower systolic blood pressures in patients with pulmonary hypertension [11]. Also, there was a significant difference in some echocardiographic characteristics between patients with measurable pulmonary artery pressure and those without measurable pulmonary artery pressure. The differences in echocardiographic findings in heart failure patients with and without pulmonary hypertension have been inconsistent [11,12]. Patients with measurable pulmonary artery pressure in our study had lower E-wave Deceleration time (ms) and higherE/A ratio. Amadi *et al*. also reported an increasing pulmonary artery pressure with increasing severity of diastolic dysfunction in heart failure patients [12]. This suggests that patients with worsening diastolic function may have a higher prevalence of PH. Neuman *et al*. reported an association between the severity and grade of diastolic dysfunction and estimated pulmonary arterial pressure after analyzing 477 consecutive echocardiographic studies in subjects with HFpEF [21]. In the present report, close to 70% of the patients with measurable PH had severe pulmonary hypertension. This was higher than reported by Karaye *et al*. who had a prevalence of severe PH of 22.64% [11]. The higher proportion of severe PH in our study can be partly explained by the higher proportion of cor pulmonale. The factors that were likely associated with moderate-to-severe pulmonary hypertension in our study were female sex, right heart failure, and right atrial dilation. After adjusting for other variables, right atrial dilatation was independently associated with pulmonary hypertension.

Studies have shown that pulmonary hypertension is associated with increased morbidity and mortality in patients with heart failure [17,22]. The case fatality in our study was 10.6% with all deaths occurring in patients with moderate-to-severe pulmonary hypertension. It is difficult to draw strong conclusions from this since we did not compare mortality between patients with and without pulmonary hypertension. In a study that followed up patients with heart failure for 6 months, mortality was significantly higher in patients with pulmonary hypertension (30.4%) compared to patients without pulmonary hypertension (8.6%) [23]. Even among outpatients with heart failure followed for several months, pulmonary hypertension is associated with high mortality [9]. A recent study in Kenya reported that severe pulmonary hypertension was significantly associated with mortality in a heterogeneous population with PH [19].

### Limitation

This study has some limitations. Firstly, the pulmonary pressures reported in this study were those obtained upon admission, using echocardiography. Right heart catheterization is the gold standard for measuring pulmonary pressure, but there is a good correlation between pulmonary pressure measured by right heart catheterization and by echocardiography. The pulmonary artery pressure was not re-evaluated when patients were stabilized and before discharge. This might have resulted in a lower prevalence of PH or decreased severity of PH. Also, we had only 4 patients without PH versus 62 with PH, so it was not possible to compare patients with and without PH. Thus we had to compare those with measurable and non-measurable pulmonary artery pressure. The fact that the pulmonary pressure was not measurable using a tricuspid regurgitation jet in part of the cohort did not rule out PH in these patients.

## Conclusion

The prevalence of pulmonary hypertension in hospitalized heart failure patients was high with two-thirds of the patients having severe PH, and most commonly occurred in females. All deaths occurred in patients with moderate-to-severe PH.

### What is known about this topic

- Pulmonary hypertension is a common and severe complication in patients with heart failure and it is associated with poor outcomes.

### What this study adds

- There was a high prevalence of pulmonary hypertension in hospitalized patients with decompensated heart failure in Buea, Cameroon;
- Pulmonary hypertension was severe in more than half of patients with pulmonary hypertension;
- All deaths occurred in patients with pulmonary hypertension.

## References

[1] Damasceno A, Cotter G, Dzudie A, Sliwa K, Mayosi BM (2007). Heart failure in sub-Saharan Africa: time for action. J Am CollCardiol.

[2] Damasceno A, Mayosi BM, Sani M, Ogah SO, Mondo C, Ojji D (2012). The causes, treatment, and outcome of acute heart failure in 1006 Africans from 9 countries. Arch Intern Med.

[3] Bigna JJ, Noubiap JJ, Nansseu JR, Aminde LN (2017). Prevalence and etiologies of pulmonary hypertension in Africa: a systematic review and meta-analysis. BMC Pulm Med.

[4] Dzudie A, Dzekem BS, Ojji DB, Kengne AP, Mocumbi AO, Sliwa K, Thienemann F (2020). Pulmonary hypertension in low-and middle-income countries with a focus on sub-Saharan Africa. Cardiovasc Diagn Ther.

[5] Hoeper MM, Humbert M, Souza R, Idrees M, Sliwa K, Jung Z (2016). A global view of pulmonary hypertension. Lancet Respir Med.

[6] Agbor VN, Essouma M, Ntusi NAB, Nyaga FU, Bigna JJ, Noubia JJ (2018). Heart failure in sub-Saharan Africa: A contemporaneous systematic review and meta-analysis. Int J Cardiol.

[7] Rosenkranz S, Gibbs JS, Wachter R, De Marco T, Vonk-Noordegraaf A, Vachiéry JL (2016). Left ventricular heart failure and pulmonary hypertension. Eur Heart J.

[8] Guazzi M, Gomberg-Maitland M, Arena R (2015). Pulmonary hypertension in heart failure with preserved ejection fraction. J Heart Lung Transplant.

[9] Ghio S, Gavazzi A, Campana C, Inserra C, Klersy C, Sebastiani R (2001). Independent and additive prognostic value of the right ventricular systolic function and pulmonary artery pressure in patients with chronic heart failure. J Am Coll Cardiol.

[10] Aronson D, Darawsha W, Atamna A, Kaplan M, Makhoul FB, Mutlak D (2013). Pulmonary hypertension, right ventricular function, and clinical outcome in acute decompensated heart failure. J Card Fail.

[11] Karaye KM, Saidu H, Bala MS, Yahaya IA (2013). Prevalence, clinical characteristics, and outcome of pulmonary hypertension among admitted heart failure patients. Ann Afr Med.

[12] Amadi VN, Ajayi EO, Akintomide OA, Abiodun OO, Bamikole JO, Balogun OM (2016). Pulmonary Hypertension in Heart Failure Patients Presenting at OAUTHC, Ile-Ife, Nigeria. Clinical Medicine Insights: Cardiology.

[13] Galiè N, Humbert M, Vachiery JL, Gibbs S, Lang I, Torbicki A (2016). 2015 ESC/ERS Guidelines for the Diagnosis and Treatment of Pulmonary Hypertension. Eur Heart J.

[14] Guazzi M, Borlaug BA (2012). Pulmonary Hypertension Due to Left Heart Disease. Circulation.

[15] Vachiéry JL, Adir Y, Barberà JA, Champion H, Coghlan JG (2013). Pulmonary Hypertension Due to Left Heart Diseases. J. Am. Coll. Cardiol.

[16] Thienemann F, Dzudie A, Mocumbi AO, Blauwet L, Sani UM, Karaye MK (2016). The causes, treatment, and outcome of pulmonary hypertension in Africa: insights from the pan African pulmonary hypertension cohort (PAPUCO) Registry. Int J Cardiol.

[17] Dzudie A, Kengne AP, Thienemann F, Sliwa K (2014). Predictors of hospitalizations for heart failure and mortality in patients with pulmonary hypertension associated with left heart disease: a systematic review. BMJ Open.

[18] Nkoke C, Jingi AM, Aminde LN, Teuwafeu D, Nkouonlack C, Noubiap JJ (2019). Heart failure in a semi-urban setting in Cameroon: clinical characteristics, etiologies, treatment, and outcome. J Xiangya Med.

[19] Ngunga M, Mansur Abeid A, Mohamed J, Barasa A (2020). Long-Term Outcomes and Factors Associated with Mortality in Patients with Moderate to Severe Pulmonary Hypertension in Kenya. Glob Heart.

[20] Bursi F, McNallan SM, Redfield MM, Nkomo VT, Lam CSP, Weston SA (2012). Pulmonary pressures and death in heart failure: a community study. J Am CollCardiol.

[21] Neuman Y, Kotliroff A, Bental T, Siegel RJ, David D, Lishner M (2008). Pulmonary artery pressure and diastolic dysfunction in normal left ventricular systolic function. Int J Cardiol.

[22] Szwejkowski BR, Elder DH, Shearer F, Jack D, Choy AM (2012). Pulmonary hypertension predicts all-cause mortality in patients with heart failure: A retrospective cohort study. Eur J Heart Fail.

[23] Aronson D, Eitan A, Dragu R, Burger AJ (2011). Relationship between reactive pulmonary hypertension and mortality in patients with acute decompensated heart failure. Circ Heart Fail.

